# Targeting Checkpoint Receptors and Molecules for Therapeutic Modulation of Natural Killer Cells

**DOI:** 10.3389/fimmu.2018.02041

**Published:** 2018-09-10

**Authors:** Nayoung Kim, Hun Sik Kim

**Affiliations:** ^1^Department of Convergence Medicine, University of Ulsan College of Medicine, Seoul, South Korea; ^2^Asan Institute for Life Sciences, Asan Medical Center, University of Ulsan College of Medicine, Seoul, South Korea; ^3^Department of Biomedical Sciences, University of Ulsan College of Medicine, Seoul, South Korea; ^4^Department of Microbiology, University of Ulsan College of Medicine, Seoul, South Korea; ^5^Stem Cell Immunomodulation Research Center (SCIRC), Asan Medical Center, University of Ulsan College of Medicine, Seoul, South Korea

**Keywords:** NK cells, immune checkpoints, checkpoint blockade, combined targeting, cancer immunotherapy

## Abstract

Among the most promising therapeutic modalities for cancer treatment is the blockade of immune checkpoint pathways, which are frequently co-opted by tumors as a major mechanism of immune escape. CTLA-4 and PD-1 are the representative examples, and their blockade by therapeutic antibodies leads to enhanced anti-tumor immunity with durable clinical responses, but only in a minority of patients. This has highlighted the need to identify and target additional immune checkpoints that can be exploited to further enhance immune responses to refractory cancers. These emerging targets include natural killer (NK) cell-directed checkpoint receptors (KIR and CD94/NKG2A) as well as the NK- and T cell-expressed checkpoints TIM-3, TIGIT, CD96, and LAG-3. Interestingly, the potentiation of anti-tumor immunity by checkpoint blockade relies not only on T cells but also on other components of the innate immune system, including NK cells. NK cells are innate lymphoid cells that efficiently kill tumor cells without MHC specificity, which is complementary to the MHC-restricted tumor lysis mediated by cytotoxic T cells. However, the role of these immune checkpoints in modulating the function of NK cells remains unclear and somewhat controversial. Unraveling the mechanisms by which these immune checkpoints function in NK cells and other immune cells will pave the way to developing new therapeutic strategies to optimize anti-tumor immunity while limiting cancer immune escape. Here, we focus on recent findings regarding the roles of immune checkpoints in regulating NK cell function and their potential application in cancer immunotherapy.

## Introduction

Natural killer (NK) cells express an array of inhibitory receptors, such as killer immunoglobulin (Ig)-like receptors (KIRs), CD94/NKG2A, programmed cell death protein 1 (PD-1), cytotoxic T-lymphocyte-associated protein 4 (CTLA-4), T cell immunoglobulin- and mucin-domain-containing molecule 3 (TIM-3), T cell immunoreceptor with Ig and immunoreceptor tyrosine-based inhibition motif (ITIM) domains (TIGIT), CD96, and lymphocyte activation gene 3 (LAG-3) (1–5). The primary mechanism of NK cell activation is governed by the “missing-self hypothesis.” NK cells do not attack healthy cells when their inhibitory receptors (KIRs and CD94/NKG2A on human NK cells and Ly49 family members on mouse NK cells) are engaged by MHC class I molecules on target cells, but downregulation of MHC class I, as frequently occurs in virally transformed or neoplastic cells, results in NK cell activation (6). In addition, activation of resting NK cells is rarely triggered by the ligation of a single activating receptor (2). Instead, effective cytotoxicity against tumor cells requires co-engagement of specific activating receptors or pre-activation by cytokines (e.g., IL-2 or IL-15) (7). This additional checkpoint is mediated by common signaling molecules [e.g., c-Cbl, glycogen synthase kinase (GSK)-3β, diacylglycerol kinase (DGK)ζ, or cytokine-inducible Src homology-2 (SH2)-containing protein (CIS)] downstream of diverse activating receptors (1), which provides an additional strategy to enhance NK cell reactivity against tumor cells. Since landmark publications have shown the significant clinical efficacy of PD-1 and/or CTLA-4 blockade in patients with melanoma and other non-treatable cancers (8, 9), much attention has been drawn to immune checkpoint receptors and their cognate ligands. Here, we focus on inhibitory receptors that serve as checkpoints in human NK cell activation, focusing on the key signaling pathways mediated by these receptors and their clinical relevance.

## Immune checkpoint receptors

### KIR and CD94/NKG2A

**Figure 1:**
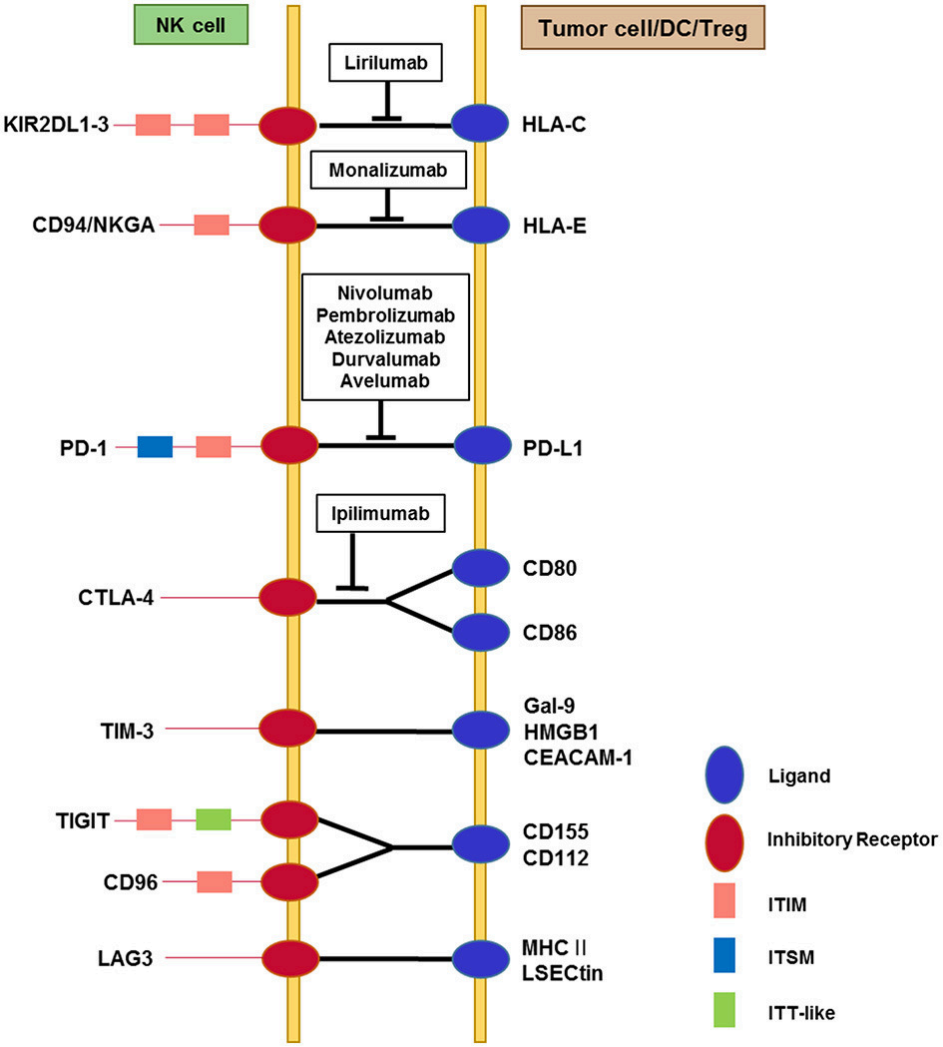
Interactions among immune checkpoint receptors and ligands affecting NK cell function. NK cells express multiple immune checkpoint receptors, which can interact with their cognate ligands on tumor cells or on other immune cells, particularly dendritic cells and Tregs. The red circles represent immune checkpoint receptors and the blue circles represent ligands. The pink squares represent classical ITIM motifs and the light blue squares represent ITSM motifs, both of which mediate inhibitory signals. TIGIT contains an ITT-like motif in addition to the ITIM motif in its cytoplasmic tail. Phosphorylation of the ITT-like motif upon ligand binding plays a critical role in inhibitory signaling via the recruitment of SHIP-1. Cytoplasmic domains of other immune checkpoint receptors contain less well-known motifs (not shown). TIM-3 contains five conserved tyrosine residues in the cytoplasmic tail, among which Y256 and Y263 in mouse (Y265 and Y272 in human) are phosphorylated upon ligand binding. This triggers the dissociation of Bat3 from the cytoplasmic tail of TIM-3, thereby promoting TIM-3-mediated T cell inhibition via the recruitment of Fyn to the same region in place of Bat3. LAG-3 contains a unique KIEELE motif in its cytoplasmic tail that is indispensable for the inhibitory function of LAG-3 in effector CD4^+^ T cells. Blocking antibodies that target immune checkpoints and are being developed for clinical use are displayed in the boxes.

The KIR family molecules include inhibitory KIRs, which have long cytoplasmic tails harboring two ITIMs (Figure 1), as well as activating KIRs that interact with DAP12 or FcRγ (10). The inhibitory KIRs are KIR2DL1, KIR2DL2, KIR2DL3, KIR2DL5, KIR3DL1, KIR3DL2, and KIR3DL3, and these receptors recognize HLA-A, -B, or -C. They have highly polymorphic Ig domains that confer specificity for HLA molecules (11). CD94/NKG2A, a heterodimeric inhibitory receptor related to C-type lectins, recognizes HLA-E, while CD94/NKG2C is an activating receptor. NKG2A, but not CD94, has two ITIMs in its cytoplasmic tail (Figure 1). The ITIMs are phosphorylated upon receptor ligation and recruit the tyrosine phosphatases SH2 domain-containing phosphatase (SHP)-1 and SHP-2 (12, 13). SHP-1 dephosphorylates Vav1, a critical mediator downstream of various activating receptors on NK cells (14, 15). Crk phosphorylation also contributes to the inhibition of NK cells following ligation of NKG2A by HLA-E (16). ITIM-based inhibition is dominant over activation in NK cells. Recruitment of SHP-1 by ITIM-bearing receptors appears to inhibit signaling at a proximal step, such that most downstream signals are blocked (2). Interaction of NK cell inhibitory receptors with MHC I ligands on target cells results in complete inhibition of polarization and release of cytotoxic granules (17). Besides their inhibitory function, the interaction of KIRs with MHC I ligands during NK cell development is crucial for their education against self-recognition (2, 18, 19). Accordingly, NK cells can maintain their intrinsic responsiveness against MHC I-deficient target cells, a process referred to as licensing.

As tumor cells exhibit variable expression of MHC I ligands, adoptive transfer of alloreactive NK cells has emerged as a promising strategy that overcomes this checkpoint and creates a condition of “missing-self” recognition. Some solid tumors and leukemias/lymphomas also use the upregulation of HLA-E to evade killing by NK and T cells (20–22). In this respect, another approach that mimics missing-self recognition is treatment with blocking antibodies against KIRs and/or NKG2A on autologous NK cells. Lirilumab (IPH2102) and monalizumab (IPH2201) are IgG4 monoclonal antibodies (mAbs) currently in clinical development that target KIR2DL1-3 and NKG2A, and antagonize the inhibition of NK cells mediated by HLA-C and HLA-E on tumor cells, respectively (3, 23). The anti-KIR antibody (IPH2101) had acceptable safety without significant toxicity or autoimmunity in multiple myeloma (MM) and acute myeloid leukemia (AML) patients (24, 25). IPH2101 treatment enhanced *ex vivo* NK cell cytotoxicity in MM patients, but did not increase NK cell numbers or cytotoxicity in AML patients. A phase II clinical trial of lirilumab in MM was stopped due to a lack of efficacy, presumably because of decreased responsiveness of KIR2D+ NK cells, accompanied by a loss of KIR2D expression (26). As pan-KIR2D blockade with IPH2101 as a monotherapy was not effective (26, 27), it is currently being widely tested in combination with other therapeutics, including lenalidomide, tumor-targeting monoclonal antibodies (mAbs) such as elotuzumab (an anti-SLAMF7 antibody) or rituximab (an anti-CD20 antibody), and other forms of immune checkpoint blockade (3, 28–31). MM cells upregulate MHC class I; thus blocking inhibitory KIRs could enhance the anti-tumor effect of NK cells in combination with lenalidomide, which is currently used with steroids (28). In combination with anti-CD20 mAbs, anti-KIR treatment (IPH2101) enhances NK cell-mediated, rituximab-dependent cytotoxicity against lymphoma *in vitro* and *in vivo* in KIR transgenic and syngeneic murine lymphoma models (29). Elotuzumab has also been developed to target MM in combination with other therapies, although it has no single-agent activity in advanced MM (32). Monalizumab improves NK cell dysfunction in chronic lymphocytic leukemia (CLL) (33). Moreover, multiple studies demonstrated the expression of NKG2A on tumor-infiltrating NK and T cells in various cancers, including breast cancer (34), cervical cancer (35), lung cancer (36), and hepatocellular carcinoma (37). Given the association between HLA-E overexpression and a poor prognosis in solid tumors (37–39), these studies support NKG2A blockade as a promising strategy to enhance anti-tumor immune responses. Monalizumab is currently under clinical investigation as a single agent in ovarian cancer or in combination with cetuximab (anti-EGFR) and durvalumab (anti-PD-L1) for advanced-stage solid cancers (3, 31). Taken together, combining anti-KIR or anti-NKG2A mAbs with chemotherapy or other mAbs targeting tumor antigens or immune checkpoint molecules may be a promising strategy to achieve clinical efficacy.

### CTLA-4 and PD-1

Co-inhibitory signaling molecules are well-described for T cells, particularly in the context of cancer immunology. The most notable examples are CTLA-4 and PD-1. CTLA-4 is a key regulator of T cell expansion, while PD-1 plays an important role in regulating T cell effector function. As of March 2018, six antibodies targeting these immune checkpoint pathways have been approved for clinical use: ipilimumab (anti-CTLA-4), nivolumab (anti-PD-1), pembrolizumab (anti-PD-1), atezolizumab (anti-PD-L1), durvalumab (anti-PD-L1), and avelumab (anti-PD-L1) (40). Therapeutic strategies targeting the CTLA-4 or PD-1 pathway restore T cell function in the cancer microenvironment and lead to durable clinical responses in various cancer types (8, 41–43). Further, combined blockade of both pathways has an additive therapeutic benefit but could come at the cost of a higher rate of adverse effects (44, 45). Various combination strategies employing PD-1 and CTLA-4 blockade are currently under investigation. The therapeutic efficacy of PD-1 and/or CTLA-4 blockade is thought to rely largely on the rescue of tumor-specific T cells from exhaustion and restoration of their effector functions.

The co-stimulatory receptor CD28 and the co-inhibitory receptor CTLA-4 compete for the same ligands, CD80 (B7-1) and CD86 (B7-2; Figure 1). CTLA-4 is a structural homolog of CD28, but binds CD80/CD86 with greater avidity and affinity. Unlike many other inhibitory receptors, CTLA-4 lacks a classical signaling motif such as an ITIM in its cytoplasmic tail. CTLA-4 activates the serine/threonine phosphatase PP2A, which inhibits Akt activation without affecting PI3K activity (46). CTLA-4 is found on activated mouse NK cells, and its engagement with B7-1 inhibits IFN-γ production in response to mature dendritic cells (47). CTLA-4^+^ regulatory T cells (Tregs) suppress NK cell cytotoxicity in cetuximab-treated head and neck cancer patients (48). Of interest, in melanoma, the activity of anti-CTLA-4 antibodies is also attributed to the selective depletion of Tregs mediated by Fc receptors (49, 50). Clinical outcome of anti-CTLA-4 treatment in melanoma patients correlates with low expression of TIM-3 on circulating T and NK cells prior to and during therapy, and correlates with an increased frequency of mature circulating CD3^−^CD56^dim^CD16^+^ NK cells during treatment (51). Survival also correlates with low serum IL-15 levels, which raises a concern regarding treating cancer patients with IL-15, which may lead to the upregulation of PD-1 and TIM-3 on T and NK cells (51). However, B7.1-CD28/CTLA-4 was not required to trigger human NK cell activation in a previous study (52). Furthermore, CD28/B7 co-stimulation was not required for peripheral NK cells to control murine cytomegalovirus infection (53). Thus, it is possible to speculate that anti-CTLA-4 therapy improves NK cell function indirectly via blockade of suppressive CTLA-4^+^ Tregs (50) and/or restoration of CTLA-4^+^ T cell function (54, 55).

The ligands of PD-1 are PD-L1 (B7-H1) and PD-L2 (B7-DC), which are upregulated in diverse tumor cells (56, 57). Their engagement of PD-1 on T cells mediates potent inhibition of T cell receptor (TCR) signaling and effector functions, thus allowing tumor cells to escape immunosurveillance (58, 59). Accordingly, blockade of PD-1/PD-L1 interactions rescues PD-1^+^ T cells from exhaustion and restores their anti-tumor function (60, 61). The cytoplasmic domain of PD-1 contains one ITIM and one immunoreceptor tyrosine-based switch motif (ITSM); the latter interacts with the phosphatases SHP-1/2. Specifically, Y248 of the PD-1 ITSM associates with SHP-2 and is required for the inhibition of PI3K/Akt activation (62). In healthy humans, PD-1 is expressed on approximately one-fourth of peripheral blood NK cells (Figure 1). Its expression is confined to CD56^dim^NKG2A^−^KIR^+^CD57^+^ mature NK cells, and is not expressed on CD56^bright^ NK cells (63). PD-1^+^ NK cells are thought to be memory-like NK cells (64) or functionally exhausted, given their impaired cytotoxicity and cytokine production (65, 66). PD-1 is upregulated on NK cells from ascites of ovarian cancer patients and on peripheral blood NK cells from Kaposi sarcoma patients, which suggests impaired NK cell function (66, 67). Treatment with an anti-PD-1 antibody increases NK cell cytotoxicity against autologous MM cells *in vitro* (68). Activated primary human NK cells efficiently kill colorectal cancer cells in organoid cultures independently of PD-L1 expression (69). Tumor-associated macrophage-like monocytes suppress activation of PD-1^+^ NK cells from patients with Hodgkin's lymphoma and diffuse large B cell lymphoma, and this suppression is reversed by PD-1 blockade *in vitro* (70). Moreover, PD-1 blockade can induce the expression of genes typically involved in cytolysis and cytokine production, including IFN-γ, in T lymphocytes (71). Because these factors can also boost NK cell function, this might be an additional mechanism underlying the clinical efficacy of PD-1 antibodies, in addition to their direct effects on NK cells. In summary, PD-1 and CTLA-4 blockade may enhance the anti-tumor activity of NK cells both directly and indirectly, via other immune cells such as tumor-specific T cells.

### TIM-3

TIM-3 is an activation-induced checkpoint receptor that was originally identified on activated CD4^+^ T helper 1 (Th1) and CD8^+^ T cytotoxic 1 (Tc1) T cells (72, 73). TIM-3 is also expressed in Th17 cells and Tregs, and on diverse innate immune cells including NK cells, NKT cells, and myeloid cells (31). The expression of TIM-3 is low on resting T cells, but strongly upregulated on activated and exhausted T cells. TIM-3 is often co-expressed with PD-1 and has been implicated in T cell exhaustion during chronic viral infection and cancer (74, 75). Blockade of TIM-3 alone or in combination with PD-1 reversed T cell exhaustion and reduced tumor growth by restoring T cell effector function in several preclinical mouse models (74, 76, 77). In contrast to T cells, NK cells express TIM-3 basally, and their expression of TIM-3 is the highest among human PBMCs (Figure 1) (78). TIM-3 is expressed on all mature CD56^dim^CD16^+^ NK cells and is further upregulated upon stimulation with the cytokines IL-12, IL-15, and/or IL-18 (79, 80). In addition, cytokine activation induces TIM-3 expression on immature CD56^bright^CD16^−^ NK cells (79), suggesting TIM-3 as a marker for mature and/or activated NK cells. The cognate ligands for TIM-3 include galectin-9 (Gal-9) (81), phosphatidylserine (PtdSer) on apoptotic cells (82), high mobility group box (HMGB)1 (83), and carcinoembryonic antigen-related cell adhesion molecule (CEACAM)-1 (5, 84). TIM-3 does not have a classical signaling motif in its cytoplasmic tail such as an ITIM or ITSM. Instead, TIM-3 has five conserved tyrosine residues in its cytoplasmic tail, among which Y256 and Y263 (in mouse) are important for TIM-3 signaling through regulated interaction with HLA-B-associated transcript 3 (Bat3) (5, 85). Bat3 is bound to TIM-3 at the steady state and recruits catalytically active Lck, which can promote T cell signaling. Upon binding of TIM-3 to its cognate ligands (e.g., Gal-9 and CEACAM-1), Y256 and Y263 are phosphorylated, leading to the dissociation of Bat3, thereby promoting T cell inhibition (84, 85). Bat3 and Fyn, a Src kinase that mediates T cell anergy (86), compete for the same binding domain in TIM-3. Thus, Bat3 might be a key determinant of TIM-3 function via regulation of the recruitment of certain signaling components.

Compared to the conserved role of TIM-3 in the suppression of activated T cells, the functional role of TIM-3 on NK cells is controversial. TIM-3 engagement has been shown to have opposing effects on NK cell activation depending on the experimental design. Cross-linking of TIM-3 with an agonistic antibody significantly decreased the cytotoxicity of primary NK cells and the NK cell line NKL (79), whereas stimulation of TIM-3 via Gal-9 selectively enhanced the production of IFN-γ by NK cells (80). Nonetheless, Gal-9 can inhibit the function of human and murine NK cells independently of TIM-3 (87). TIM-3 is upregulated in peripheral blood NK cells from patients with advanced gastric cancer (88), lung adenocarcinoma (89), and advanced melanoma (90), and this sustained increase in TIM-3 expression is associated with NK cell exhaustion and dysfunction. It remains unclear whether this dysfunction of TIM-3^+^ NK cells is related to specific or multiple ligands on these cancers, and this merits further investigation. TIM-3 is also found on tumor-infiltrating NK cells in approximately 75% of patients with gastrointestinal stromal tumors (GIST) (91). Of interest, TIM-3^+^ tumor-infiltrating NK cells in GIST do not co-express PD-1 (91). However, in a mouse model using lung tumor cells (TC-1) that express human papillomavirus oncoproteins and are MHC class I-deficient, TIM-3^+^PD-1^+^ NK cells could be detected and were functionally exhausted (65). Blockade of TIM-3 on NK cells from patients with advanced melanoma and lung adenocarcinoma rescues exhausted NK cells and results in increased NK cell cytotoxicity and IFN-γ production (89, 90, 92). TIM-3 expression has also been found to correlate with advanced disease and poor prognosis. These studies suggest that TIM-3 serves as a prognostic biomarker for cancer and is a potential therapeutic target to restore NK cell reactivity against cancer. However, TIM-3 blockade reduces NK cell-mediated killing of pancreatic cancer cell lines (93), and blocking Gal-9 reduces IFN-γ production by NK cells from healthy donors upon incubation with primary AML blasts (94). The promiscuous binding of TIM-3 to multiple ligands may account for its controversial effects on NK cell function. In summary, given the conflicting effects of TIM-3 modulation on NK cell function, further studies will be necessary to determine the precise role of TIM-3 in cancer surveillance by NK cells and to better harness the therapeutic potential of TIM-3 blockade in NK cell-mediated cancer therapy.

### TIGIT and CD96

TIGIT and CD96 are inhibitory receptors that compete with an activating receptor DNAM-1 (CD226) for binding to nectin and nectin-like ligands (e.g., CD155 and CD112; Figure 1) (4). CD155 is the main ligand for TIGIT and CD96, and is highly expressed on many types of tumor cells (95–97). TIGIT contains ITIM and immunoreceptor tyrosine tail (ITT)-like motifs in its cytoplasmic tail. ITT-like motifs play an important role in mediating inhibitory signaling (98, 99). Engagement of TIGIT by CD155 induces its phosphorylation through Fyn and Lck, resulting in recruitment of SHIP-1, which downregulates the PI3K, MAPK, and NF-κB signaling pathways (5). The cytoplasmic tail of CD96 has an ITIM-like motif for inhibitory signaling, but human CD96 differs from mouse CD96 by the presence of a YXXM motif, similar to that found in activating receptors (e.g., NKG2D and CD28).

TIGIT is readily detectable on resting human NK cells but not on mouse NK cells, and is upregulated upon NK cell activation (4, 100). By contrast, CD96 is constitutively expressed on both resting human and mouse NK cells (4, 101). Engagement of TIGIT by CD155 inhibits human NK cell cytotoxicity and cytokine production by counterbalancing DNAM-1-mediated activation, and this can be reversed by antibody-mediated TIGIT blockade (100, 102). TIGIT blockade also renders NK cells resistant to inhibition by myeloid-derived suppressor cells (103). CD96 binding to CD155 inhibits IFN-γ production by NK cells in mice (104). Accordingly, antibody blockade of CD96 promotes NK cell production of IFN-γ and leads to improved tumor control of lung metastases in three different mouse models, both alone or, more effectively, in combination with anti-CTLA-4, anti-PD-1, or doxorubicin (105). Combined blockade of TIGIT and PD-1 also resulted in significant tumor clearance via enhanced CD8^+^ T cell effector function (106). It remains unclear why both TIGIT and CD96 are required to counteract DNAM-1-mediated NK cell activation. One possibility is that they play a complementary role in the control of NK cell effector function; TIGIT mainly regulates cytotoxicity, whereas CD96 controls IFN-γ production, as described above. In support of this hypothesis, CD96 blockade in *Tigit*^−/−^ mice results in better control of B16F10 lung metastasis compared with wild-type mice (107), although lung metastasis is unaffected in *Tigit*^−/−^ mice (104). Differences in ligand specificity and affinity may also contribute to the net signaling outcome of these paired receptors in a context-dependent manner (4, 105). Despite efficacy in certain preclinical tumor models, whether blockade of TIGIT and/or CD96 modulates NK cell effector function and results in clinical responses in human cancer patients remains to be seen.

### LAG-3

LAG-3 is structurally similar to CD4 but binds to MHC class II molecules with a higher affinity than CD4 (108, 109). It is expressed on activated T and NK cells (Figure 1) (109). Another potential ligand for LAG-3 is LSECtin, a member of the DC-SIGN family that is expressed on many tumors and is involved in the inhibition of anti-tumor T cell responses (110). The cytoplasmic tail of LAG-3 has three unique regions that are conserved in humans and mice: the serine phosphorylation site, a KIEELE motif, and glutamic acid-proline (EP) repeats (111). Of these, the KIEELE motif is required for the inhibitory function of LAG-3 in CD4^+^ T cells. T cell effector function is inhibited by engagement of LAG-3 and is improved by LAG-3 blockade (111–113). Of interest, LAG-3 is involved in T cell exhaustion, and therefore combined blockade of LAG-3 and PD-1 synergize to restore T cell function (114, 115). However, the role of LAG-3 in the regulation of NK cell function remains unclear and requires further investigation. NK cells from LAG-3-deficient mice show defects in killing of certain tumor targets, whereas lysis of MHC class I-mismatched cells was not affected by LAG-3 deletion (116). In addition, blocking the LAG-3 pathway with an anti-LAG-3 antibody or soluble LAG-3 has no effect on human NK cell cytotoxicity (117). In conclusion, LAG-3 could be a good candidate for immunotherapy because of its potential to activate both T and NK cells, but further studies on its specific role in NK cells are necessary.

## Checkpoint molecules in NK cell activation

In contrast to the MHC-restricted activation of T cells, NK cell activation does not require the recognition of specific antigen presented on MHC molecules. Rather, NK cells have an array of activating receptors with unique ligand specificity and signaling properties: receptors containing immunoreceptor tyrosine-based activation motifs (ITAMs; e.g., CD16, NKp30, and NKp46), the DAP10-associated receptor NKG2D, receptors of the signaling lymphocytic activation molecule (SLAM) family (e.g., 2B4), and other receptors (e.g., DNAM-1) (2, 118). Given the expression of multiple and heterogeneous ligands on tumor cells, it would be desirable to target common signaling molecules that restrain NK cell activation via multiple activating receptors. Modulation of these molecules, which serve as checkpoints, may provide an additional strategy to improve NK cell function. We and others recently described this class of signaling molecules, which includes c-Cbl and GSK-3β (1).

Cbl family members, including c-Cbl and Cbl-b, primarily serve as negative regulators of signaling associated with activating receptors on various lymphocytes (119). In human NK cells, knockdown of c-Cbl rather than Cbl-b augments cytotoxicity and cytokine production by NK cells via multiple activating receptors in a Vav1-dependent manner (15). In addition, c-Cbl serves as a checkpoint for NK cell activation through different activating receptors by imposing a requirement for receptor co-engagement in resting NK cells. In mouse models, Cbl-b-deficiency enhances NK cell function and results in better control of lung metastases (120). Although the therapeutic potential of Cbl-b in human NK cells requires further investigation, modulation of Cbl proteins may provide a promising therapeutic strategy to increase NK cell reactivity against tumor cells.

Using a model of NK cell activation via different activating receptors, GSK-3β was identified as a common downstream signaling molecule in NK cell activation (121). GSK-3β inhibits NK cell function, including cytotoxicity and cytokine production, because its kinase activity is critically involved in these pathways. Accordingly, knockdown or pharmacologic inhibition of GSK-3β increases NK cell function via different activating receptors, suggesting GSK-3β as a checkpoint molecule involved in diverse NK cell activation pathways. Likewise, the NK cell dysfunction observed in AML patients could be reversed by genetic or pharmacologic GSK-3β inactivation (122). Furthermore, NK cells expanded *ex vivo* in the presence of a GSK-3β inhibitor exhibit a more mature phenotype and significantly higher anti-tumor activity (123), suggesting GSK-3β as a promising therapeutic target for NK cell-based therapy.

DGKζ is a negative regulator of diacylglycerol-mediated signaling, which is triggered by diverse activating receptors. DGKζ deficiency in mice increases NK cell function in an extracellular-related kinase (ERK)-dependent manner (124). DGKζ-deficient mice reject tumors more efficiently *in vivo*, although the deficiency does not affect the expression or function of NK cell inhibitory receptors. DGKζ is expressed in macrophages and dendritic cells (DCs), where it regulates microbial recognition (125). DGKζ also limits the generation of natural Tregs by inhibiting their development (126, 127).

Two members of the suppressor of cytokine signaling (SOCS) family, CIS and SOCS2, are reported to control NK cell differentiation and activity (128). Importantly, CIS serves as a novel checkpoint in NK cell-mediated anti-tumor responses by targeting IL-15 signaling. The gene encoding CIS, *cish*, is highly induced by IL-15, and the deletion of *cish* rendered NK cells hypersensitive to IL-15 (129). *Cish*^−/−^ mice show reduced metastasis in various tumor models, likely due to upregulation of Janus kinase (JAK)-signal transducers and activators of transcription (STAT) signaling in activated NK cells.

## Conclusions and perspectives

NK cells are innate lymphoid cells with an intrinsic ability to kill diverse tumor cells without MHC restriction. Thus, NK cells are now considered promising therapeutic targets for cancer immunotherapy, particularly for the control of metastases and leukemia/lymphoma. Recent studies have demonstrated the clinical efficacy of NK cell-based therapies in the treatment of various cancer types. However, success is still limited, and there is substantial interest in identifying therapeutic targets to improve NK cell reactivity against tumor cells. Initially discovered as a safeguard mechanism to ensure self-tolerance and prevent autoimmunity, immune checkpoint receptors have been explored as attractive therapeutic targets to enhance anti-tumor immunity, including that mediated by NK cells. As many of these immune checkpoint receptors are not specific to NK cells, it will be important to determine the contribution of NK cells to the clinical benefit of blockade of these molecules. For example, the therapeutic benefit of blockade of PD-1 and CTLA-4 is largely thought to be due to actions on T cells rather than NK cells. A notable feature of targeting NK cell-specific checkpoints (e.g., inhibitory KIRs and NKG2A), alone or in combination with others (e.g., PD-1 blockade by nivolumab), is the lack of severe toxicity (3, 25), which could provide strategic flexibility for NK cell-based therapy. Checkpoint receptors often cooperate to impair T cell responses, which can be overcome by combined targeting (e.g., blockade of PD-1 and CTLA-4), resulting in improved clinical outcomes. Given the disappointing clinical efficacy of molecules targeting NK cell-specific checkpoints as a monotherapies, therapies that target both NK cells and other effector cells, such as T cells, can be pursued. Recent studies also suggest that NK cell effector function relies on the modulation of various molecular checkpoints (e.g., Cbl, GSK-3β, DGKζ, or CIS) in diverse activation pathways, which may provide an additional strategy to enhance NK cell function. Blockade of these molecular checkpoints could facilitate the activation of NK cells by lowering the activation threshold in response to activating receptors and/or cytokines. Although the therapeutic benefit of targeting these checkpoints needs to be assessed, this information will provide new therapeutic options to improve NK cell activation, possibly in combination with other therapies, for better outcomes in the clinic.

## Author contributions

Both NK and HSK conceived, wrote the manuscript, and approved it for publication.

### Conflict of interest statement

The authors declare that the research was conducted in the absence of any commercial or financial relationships that could be construed as a potential conflict of interest.
